# Pediatric Exposures to Topical Benzocaine Preparations Reported to a Statewide Poison Control System

**DOI:** 10.5811/westjem.2017.6.33665

**Published:** 2017-07-14

**Authors:** Rais Vohra, Serena Huntington, Jennifer Koike, Kevin Le, Richard J. Geller

**Affiliations:** * California Poison Control System, Valley Children’s Hospital, Madera, California; † University of California, San Francisco, School of Pharmacy, San Francisco, California; ‡ University of California San Francisco, Fresno Medical Education and Research Program, Fresno, California

## Abstract

**Introduction:**

Topical benzocaine is a local anesthetic commonly used to relieve pain caused by teething, periodontal irritation, burns, wounds, and insect bites. Oral preparations may contain benzocaine concentrations ranging from 7.5% to 20%. Pediatric exposure to such large concentrations may result in methemoglobinemia and secondarily cause anemia, cyanosis, and hypoxia.

**Methods:**

This is a retrospective study of exposures reported to a statewide poison control system. The electronic health records were queried for pediatric exposures to topical benzocaine treated at a healthcare facility from 2004 to 2014. Cases of benzocaine exposure were reviewed for demographic and clinical information, and descriptive statistical analysis was performed.

**Results:**

The query resulted in 157 cases; 58 were excluded due to co-ingestants, or miscoding of non-benzocaine exposures. Children four years of age and younger represented the majority of cases (93%) with a median age of 1 year. There were 88 cases of accidental/ exploratory exposure, while 6 cases resulted from therapeutic application or error, 4 cases from adverse reactions, and 1 case from an unknown cause. Asymptomatic children accounted for 75.5% of cases, but major clinical effects were observed in 5 patients. Those with serious effects were exposed to a range of benzocaine concentrations (7.5–20%), with 4 cases reporting methemoglobin levels between 20.2%–55%. Methylene blue was administered in 4 of the cases exhibiting major effects.

**Conclusion:**

The majority of exposures were accidental ingestions by young children. Most exposures resulted in minor to no effects. However, some patients required treatment with methylene blue and admission to a critical care unit. Therapeutic application by parents or caregivers may lead to adverse effects from these commonly available products.

## INTRODUCTION

Topical benzocaine is a local anesthetic preparation commonly used to relieve pain caused by burns, wounds, insect bites, teething, and mouth or gum irritation. Over-the-counter topical benzocaine is marketed for teething pain in pediatric patients despite the American Academy of Pediatrics’ recommendation against its use.^1^ The widespread utilization of these products can lead to misuse by caregivers or exploratory exposures by children themselves. Benzocaine preparations used in teething (e.g. Orajel^TM^ and Anbesol^TM^) have concentrations ranging from 7.5–20%, which may lead to significant adverse effects.^2^ As a potent inducer of oxidative stress, benzocaine can result in methemoglobinemia and secondarily cause cyanosis, dyspnea, syncope, seizures, and coma.^3^–^4^ The broad availability and popular use of topical benzocaine preparations is a public health risk, especially in the pediatric population and in those with poor dental hygiene or follow-up dental care.

Prior literature related to benzocaine teething preparations is limited to a few case reports and one retrospective review.^5^–^11^ Case reports or series have previously documented incidents involving children becoming cyanotic and subsequently having elevated methemoglobin levels after benzocaine use and accidental ingestions.^6^–^11^

Some studies have documented potentially lethal methemoglobin levels with benzocaine exposure, some as high as 69%. The most common setting for exposure was benzocaine gel being administered to children for teething pain or application of “burn cream” applied to superficial burns. We conducted a systematic review of pediatric benzocaine exposures reported to a statewide poison control system.

## METHODS

This was a retrospective study performed at the California Poison Control System (CPCS). We queried electronic medical records of the CPCS for all calls from January 2004 to December 2014 associated with benzocaine using unique substance codes created by the American Association of Poison Control Center (AAPCC) to track benzocaine exposures. Data were abstracted by two researchers, and a kappa score greater than 0.7 on 10% of the data was established prior to subsequent data abstraction.

The inclusion criteria were all cases involving exposures to topical benzocaine in patients less than 18 years old who presented to a healthcare facility. We excluded cases if they were information calls, non-human exposures, non-healthcare facility exposures, exposures to non-benzocaine products, and exposures occurring with other co-ingestants. Data abstraction consisted of both demographic and clinical outcomes, including the following: age, gender, amount of benzocaine ingested, the concentration of the benzocaine product, adverse effects, presence of methemoglobinemia, interventions received, and the patient’s disposition or highest level of care provided within the healthcare facility. We used descriptive analysis and frequencies to characterize the study population and clinical outcomes related to topical benzocaine exposures.

## RESULTS

The CPCS received 157 reported benzocaine exposure cases in children less than 18 years of age who presented to a healthcare facility from January 2004 to December 2014. Of those cases, 58 met exclusion criteria, leaving 99 cases for subsequent data analysis. Patient ages ranged from one month to 12 years (median age, one year) with 93% of patients under the age of four years. Males represented the majority (56%, n=55) of exposures.

Most cases were caused by unintentional exposures related to exploratory behavior in toddlers (88.9%, n=88). Therapeutic error was the cause of six cases (benzocaine concentration higher than indicated or increased frequency of application), while four cases were considered to be “allergic reactions” and one case had an unknown cause/intent. Allergic-reaction signs and symptoms were considered as a type of adverse drug reaction, and findings recorded as described by the treating team. Route of exposure was primarily ingestions (n=88), with four dermal exposures, four cases of both ingestion and dermal exposure, two ocular exposures, and one case involving an undetermined route of exposure. In 92 exposures, the benzocaine product was used for oral indications. The majority of exposures (95%) occurred within the patients’ homes with three cases occurring at a daycare facility, one case occurring at a healthcare facility, and one case occurring in a public space. Benzocaine concentrations ranged from 7.5%–20%, with 32 cases involving the 20% formulation, 21 cases involving the 7.5% formulation, 14 cases involving the 10% formulation, and 31 cases with an unknown formulation.

Outcomes and adverse effects reported in these exposures are summarized in Table. Ninety cases resulted in no effect or minor effect (75.5% and 16.3%, respectively). Of these 90 cases, 73 patients were treated and released from the emergency department (ED), 13 presented to the ED but were lost to follow-up, two were evaluated by their primary care provider, and two had an unknown disposition. All three cases with moderate effects were treated in the ED, with two of the cases ultimately released from the ED and the remaining case lost to follow-up after presentation. One patient of the moderate-effects group required ocular irrigation, while the rest were observed and discharged. Of the five cases with major effects, two patients were admitted to an intensive care unit, two were admitted to the medical floor, and one was treated and released from the ED.

Methemoglobin concentration was measured in seven patients, and ranged from 1%–55%, with an average measured value of 24%. Methemoglobin concentration was reported in four of the cases with major effects: 20.2%, 40%, 48%, and 55%. All four cases with documented elevated methemoglobin concentration received intravenous (IV) methylene blue and supplemental oxygen. Four out of the five cases with major effects involved parents or caregivers administering the benzocaine product to the child.

## DISCUSSION

This large case series of benzocaine exposures reported to a statewide poison control system suggests that the wide availability of topical benzocaine products marketed towards pediatric-age populations continues to pose a child health hazard.

In 2000, Spiller. et al published a retrospective review of oral benzocaine exposures involving four regional poison centers.^5^ They found only minor effects associated with benzocaine exposure, and only one child with a methemoglobin concentration greater than 1%. By contrast, our study demonstrates a higher rate of complications and hospitalization rates in a large cohort of affected children. Although most of our cases were treated and released from the ED with minimal complications, a few patients had major adverse effects, mainly related to methemoglobinemia. Most cases in this study were the result of unintentional exposures to benzocaine products; however, the majority of cases showing major effects were due to intentional administration by a caregiver or parent. Besides the effects related to methemoglobinemia such as dyspnea, cyanosis, and tachycardia, there were also reports of irritant effects (e.g. vomiting and skin, throat, and ocular irritation) following exposures.

Previous reports have documented severe methemoglobinemia in pediatric patients following parental application of an oral benzocaine product. Chung et al. described a six-year-old child who presented to the ED with a methemoglobin concentration of 69.9% after being administered benzocaine gel for a toothache.^7^ Bong et al. shared a case study of a 15-month-old toddler with a complex medical history who developed a methemoglobin concentration of 42.5% after appropriate application of benzocaine gel for teething.^8^ Both children were successfully treated with IV methylene blue and oxygen therapy.

There are also reports of parent-administered dermal exposures to benzocaine. Eldadah and Fitzgerald described the case of a two-year-old child who presented with severe methemoglobinemia requiring intubation and IV methylene blue after parental application of a benzocaine cream to a rash.^10^ Poredos et al. shared a case report of a four-year-old child with deep dermal and subdermal burns who was administered a 1.2% benzocaine cream, and subsequently developed cyanosis and lethargy with a methemoglobin level of 13%.^7^ Although dermal exposures to benzocaine products were not prevalent in our study, it is prudent to acknowledge the common theme—a high incidence of toxicity following parental or caregiver application.

In April 2011 the United States Food and Drug Administration (FDA) released a drug safety communication regarding the potential for serious side effects, including methemoglobinemia, associated with the use of topical benzocaine products.^12^ Our data showed a decline in the number of benzocaine exposure cases called into the CPCS in the years following the release of the FDA drug safety communication (Figure). However, this study indicates that exposures are still occurring, underscoring the need for further education of parents and caregivers regarding the appropriate pediatric indications and application instructions for benzocaine-containing products. Restricting these products at the retail level, perhaps by placing them “behind the counter” at pharmacies and related vendors, may also help prevent overuse of these products and alert parents and caregivers about their risks.

## LIMITATIONS

This study has several limitations. Many of the pediatric benzocaine cases called into the CPCS from 2004–2014 did not meet inclusion criteria. This discrepancy may be the result of substance misclassification upon initial data entry at the time of the phone call. A second limitation of the study is the exclusion of well-appearing or asymptomatic exposed patients who were managed at home. Per CPCS guidelines, many children were managed at home if they were asymptomatic following exposures. For this study, which was designed to identify trends with the most critical cases, we chose to focus only on those patients who were treated at healthcare facilities in order to better characterize the extent of severe reactions following an exposure. Analyzing all benzocaine exposures (home + healthcare facility) would have given a larger sample size and perhaps a more complete picture of the problem.

As a retrospective review, many variables could not be controlled in this study, and the data collected were not recorded with our study parameters in mind, making it impossible to draw conclusions regarding causation. One particular drawback to this study is that information was incomplete due to undercoding of clinical signs and symptoms at the time of poison control assessment. More thorough information regarding the amount ingested, product concentration and patient disposition, perhaps collected prospectively, could have allowed for a more detailed characterization of benzocaine exposures.

## CONCLUSION

The availability of topical benzocaine preparations over the counter poses a challenge to pediatric patient safety, as parents or caregivers may not be fully informed regarding the hematologic risks associated with benzocaine toxicity. To reduce the incidence of topical benzocaine toxicity in children, the general public and clinicians treating children should be made aware about appropriate clinical indications, safe concentrations and doses, and application instructions relevant to these products. More rigorous regulations at the commercial retail level, as with ephedrine-based decongestants, may also help curb adverse reactions to these products.

## Figures and Tables

**Figure 1:**
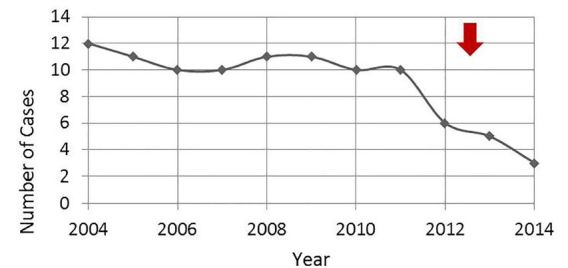
Benzocaine cases called into the CPCS from 2004 through 2014 classified by year. The red arrow indicates the publication of the FDA drug safety communication regarding benzocaine toxicity. *CPCS*, California Poison Control System; *FDA*, Food and Drug Administration.

**Table:** Adverse effects following over-the-counter benzocaine gel exposure in children.

Outcome and clinical effects	Number of cases (n=99)†
No effect	74
Minor effect	16
Drowsiness	6
Vomiting	3
Reported wheezing/trouble breathing (normal upon MD exam)	2
Swollen cheek	1
Blisters/erythema on cheek	1
Cough	1
Cyanosis (normal upon MD exam)	1
Ocular pain/irritation	1
Tachycardia	1
Hypertension	1
Moderate effect	3
Corneal abrasion	1
Difficulty breathing	1
Cyanosis	1
Lethargic	1
Vomiting	1
Major effect	5
Elevated methemoglobin levels	4
O2 saturation < 90%	3
Cyanosis	3
Seizure	1
Metabolic acidosis	1

† The outcome of one case was lost to follow-up.
